# Complete Genome Sequences of 61 Mycobacteriophages

**DOI:** 10.1128/genomeA.00389-16

**Published:** 2016-07-07

**Authors:** Graham F. Hatfull, Science Education Alliance Phage Hunters Advancing Genomics and Evolutionary Science (SEA-PHAGES) Program, KwaZulu-Natal Research Institute for Tuberculosis and HIV (K-RITH) Mycobacterial Genetics Course, University of California–Los Angeles Research Immersion Laboratory in Virology, Phage Hunters Integrating Research and Education (PHIRE) Program

**Affiliations:** a Pittsburgh Bacteriophage Institute and Department of Biological Sciences, University of Pittsburgh, Pittsburgh, Pennsylvania, USA; b Howard Hughes Medical Institute, Chevy Chase, Maryland, USA; c K-RITH, Durban, South Africa; d Department of Microbiology and Immunology, Albert Einstein College of Medicine, Bronx, New York, USA; e Department of Microbiology, Immunology, and Molecular Genetics, University of California–Los Angeles, Los Angeles, California, USA

## Abstract

Mycobacteriophages—viruses of mycobacteria—provide insights into viral diversity and evolution as well as numerous tools for genetic dissection of *Mycobacterium tuberculosis*. Here we report the complete genome sequences of 61 mycobacteriophages newly isolated from environmental samples using *Mycobacterium smegmatis* mc^2^155 that expand our understanding of phage diversity.

## GENOME ANNOUNCEMENT

Bacteriophages are the most numerous biological entities on the planet, with a global population of 10^31^ particles. With an estimated 10^23^ productive infections per second worldwide, the population is vast, dynamic, and genetically diverse (1–4). As of March 2016, the National Center for Biotechnology Information (NCBI) lists 1,757 *Caudovirales* genomes, 318 of which infect *Mycobacterium* hosts. Previous comparative analyses of mycobacteriophages revealed substantial diversity and mosaic architectures resulting from nonhomologous recombination. Integrated research-education programs such as Phage Hunters Integrating Research and Education (PHIRE) (5), Science Education Alliance Phage Hunters Advancing Genomics and Evolutionary Science (SEA-PHAGES) (6), the Mycobacterial Genetics Course at the University of Kwazulu-Natal (K-RITH), and the University of California–Los Angeles’s Research Immersion Laboratory in Virology, isolated, sequenced, and annotated the phages reported here (Table 1) using *M. smegmatis* as a host.

**TABLE 1:** Newly sequenced mycobacteriophage genomes

Phage name	Cluster	Genome (bp)	G+C content (%)	GenBank accession no.	Finding/annotating institution
Alvin	A1	49,577	63.5	KP027205	University of Pittsburgh^a^
ArcherNM	A2	52,561	64.2	KU761559	Washington State University, University of Florida^b^
Artemis2UCLA	A6	52,344	61.4	KF560333	University of California Los Angeles^d^
Bactobuster	A2	52,129	63.1	KU568494	North Carolina A&T State University^b^
Bernardo	B3	68,196	67.4	KF493879	University of California–Los Angeles^d^
Bipper	Y	77,832	67.3	KU728633	University of Pittsburgh, Florida Gulf Coast University^b^
Bricole	M1	81,128	61.6	KT591491	Old Dominion University^b^
Bruin	E	74,210	63.0	KF562099	University of California–Los Angeles^d^
Brusacoram	P	47,618	67.0	KT347313	College of St. Scholastica^b^
Carcharodon	N	43,680	66.2	KM588359	Jacksonville State University^b^
Cedasite	G1	41,901	66.6	KT355472	Morehouse College^b^
Chandler	B3	69,450	67.5	KP027207	University of Pittsburgh^a^
CloudWang3	A6	52,873	61.4	KF560332	University of California–Los Angeles^d^
Conspiracy	A5	50,755	60.6	KF560330	University of California–Los Angeles^d^
Cosmo	V	78,229	56.8	KP027195	University of KwaZulu-Natal^c^
Eidsmoe	A9	52,946	62.5	KU716094	Illinois Wesleyan University^b^
Enkosi	K1	59,052	67.2	KT281789	University of KwaZulu-Natal^c^
Glass	B2	67,509	69.0	KT880194	Hope College^b^
Graduation	A1	52,823	63.5	KF560331	University of California–Los Angeles^d^
HanShotFirst	A1	52,390	63.8	KF493880	University of California–Los Angeles^d^
HufflyPuff	E	76,323	63.0	KF562100	University of California–Los Angeles^d^
HyRo	C1	153,714	64.7	KT281790	University of KwaZulu-Natal^c^
Iracema64	A4	51,637	64.0	KU055616	La Salle University^b^
JAMaL	B4	70,841	68.8	KF493881	University of California–Los Angeles^d^
JenCasNa	A3	50,877	64.0	KU255188	Howard Hughes Medical Institute^b^
Jovo	A5	51,319	60.8	KF493882	University of California–Los Angeles^d^
Kimberlium	F1	56,826	61.4	KR935214	Gettysburg College^b^
LadyBird	A2	53,141	63.5	KT588442	St. Edward’s University^b^
Lolly9	L3	75,816	59.3	KT281791	University of KwaZulu-Natal^c^
Lumos	L3	75,586	59.3	KT372003	Indian River State College^b^
MichelleMyBell	N	42,240	66.0	KF986246	Nyack College^b^
Mosby	E	74,533	63.1	KF493883	University of California–Los Angeles^d^
Nala	E	75,894	63.1	KF562101	University of California–Los Angeles^d^
NaSiaTalie	A2	52,920	63.4	KU297783	Howard Hughes Medical Institute^b^
Numberten	B1	68,607	66.5	KJ194583	University of Pittsburgh^a^
Panchino	N	43,516	65.9	KU935727	Lincoln University^b^
Phamished	B1	68,515	66.5	KR816508	Gettysburg College^b^
PhatBacter	E	76,217	63.0	KF562102	University of California–Los Angeles^d^
Phatniss	F1	57,293	61.3	KT279576	Johns Hopkins University^b^
Phrann	N	44,872	66.3	KU935731	Southern Connecticut State University^b^
Pioneer	A9	53,219	62.6	KT285706	Indian River State College^b^
Pipsqueaks	N	43,679	66.3	KU935730	College of Charleston^b^
PopTart	F1	55,094	61.6	KT281792	University of KwaZulu-Natal^c^
Potter	B1	68,327	66.5	KU867907	University of Kansas^b^
Romney	A4	51,370	63.9	KU867906	Seton Hill University^b^
Sbash	I2	55,832	65.6	KP027201	University of KwaZulu-Natal^c^
Seabiscuit	A1	51,781	63.7	KJ194585	University of Pittsburgh^a^
Seagreen	F1	57,766	61.8	KT281793	University of KwaZulu-Natal^c^
SkinnyPete	N	43,478	66.4	KU935729	Virginia Commonwealth University^b^
Snenia	L3	75,626	59.3	KT281794	University of KwaZulu-Natal^c^
Sparkdehlily	F1	56,275	61.2	KT895280	James Madison University^b^
Tasp14	A1	51,409	63.9	KT326768	Ohio State University^b^
Texage	A3	50,081	64.0	KT326767	Merrimack College^b^
TheloniousMonk	A1	52,055	63.6	KT363731	Western Kentucky University^b^
Tres	B2	67,349	68.9	KT365402	James Madison University^b^
Wooldri	A3	50,797	64.0	KT381277	Washington State University, University of Florida^b^
Xeno	N	42,395	66.8	KU935728	Southern Connecticut State University^b^
Xerxes	N	43,698	66.3	KU935726	University of Florida^b^
XFactor	F1	55,617	61.7	KT281795	University of KwaZulu-Natal^c^
Zaka	A6	52,122	61.5	KF560334	University of California–Los Angeles^d^
Zakhe101	O	69,653	65.5	KT281796	University of KwaZulu-Natal^c^

a Phage Hunters Integrating Research and Education (PHIRE) Program, University of Pittsburgh.

b Science Education Alliance-Phage Hunters Advancing Genomics and Evolutionary Science (SEA-PHAGES).

c K-RITH Mycobacterial Genetics Course.

d University of California–Los Angeles, Research Immersion Laboratory in Virology.

Phages were isolated by direct plating of filtered soil extracts or from enriched cultures, followed by plaque purification. Electron microscopy shows that 60 have siphoviral morphotypes, and HyRo is the sole member of the *Myoviridae*. Most have isometric capsids, the exceptions being Bipper, Sbash, and Zakhe101 with prolate heads. Genomic DNA was extracted from high titer lysates, sheared, and sequenced at the University of Pittsburgh, University of California–Los Angeles, the DOE Joint Genome Institute, or Virginia Commonwealth University using either Sanger, Illumina, Ion Torrent, or 454 technology. Sequence reads were assembled using Newbler (Roche) and Consed (7) and coverage depths range from 47-fold to 2,308-fold, with an average of 200-fold. Sequence assemblies revealed discrete genome ends for 52 phages, and the 9 with circularly permuted assemblies were bioinformatically linearized such that base one was assigned in accord with other mycobacteriophages. Genomes were annotated using DNA Master (http://cobamide2.bio.pitt.edu), Phamerator (8), Glimmer (9), GeneMark (10), Aragorn (11), and tRNAscanSE (12), and functions were determined using the public databases GenBank, Protein DataBase, pfamA, and phagesdb.org with BLAST (13), and HHPred (14). Genomes were assigned to clusters or subclusters as described previously (15).

Notwithstanding the large extant collection of sequenced mycobacteriophage genomes, these newly sequenced phages considerably expand our understanding of mycobacteriophage diversity. Twenty-two are members of the largest cluster, cluster A, but span 7 of the 15 subclusters. The others are broadly distributed across other clusters, including B, C, E, F, G, I, K, L, M, N, O, and P. Cosmo has substantial nucleotide sequence similarity to the singleton phage Wildcat, forming the new cluster V. The eight cluster N phages, Cedasite (G1), and Brusacoram (P) are notable in that they contain integration-dependent immunity systems in which the phage attachment site (*attP*) is located within the repressor gene (16).

As is typical of other sequenced phage genomes, functions can be assigned to only ~25% of the predicted genes, primarily those involved in virion capsid and assembly and well-conserved genes associated with DNA metabolism. Two of the cluster A genomes (Eidsmoe, ArcherNM) contain partitioning systems in place of integration cassettes; several genomes (e.g., Phrann, Xeno) encode toxin-antitoxin systems; and three encode Lsr2 homologs (Lolly9, Lumos, and Snenia).

### Nucleotide sequence accession numbers.

Nucleotide sequence accession numbers for all phages are shown in Table 1.
